# Formulation and Characterization of an Oleuropein-Enriched Oral Spray Gel: Microbiological Performance and In Ovo Histopathological Safety

**DOI:** 10.3390/pharmaceutics18020200

**Published:** 2026-02-03

**Authors:** Levent Alparslan, Samet Özdemir, Burak Karacan, Ömer Faruk Tutar, Tunay Doğan, Remzi Okan Akar, Elifnur Gizem Yıldırım, Nusret Erdoğan

**Affiliations:** 1 Department of Pharmaceutical Technology, Faculty of Pharmacy, İstinye University, Istanbul 34010, Türkiye; 2 Department of Pharmaceutical Technology, Faculty of Pharmacy, İstanbul Health and Technology University, Istanbul 34445, Türkiye; samet.ozdemir@istun.edu.tr; 3 Department of Pharmaceutical Microbiology, Faculty of Pharmacy, İstinye University, Istanbul 34010, Türkiye; burak.karacan@istinye.edu.tr; 4 Department of Pharmacy Services, Vocational School of Health Care Services, İstinye University, Istanbul 34010, Türkiye; omer.tutar@istinye.edu.tr; 5 Department of Medical Laboratory Techniques, Vocational School of Health Care Services, İstinye University, Istanbul 34010, Türkiye; tunay.dogan@istinye.edu.tr; 6 Molecular Cancer Research Center (ISUMKAM), İstinye University, Istanbul 34010, Türkiye; remzi.akar@istinye.edu.tr; 7 Department of Medical Biochemistry, Faculty of Medicine, İstinye University, Istanbul 34010, Türkiye; 8 Department of Molecular Biology and Genetics, Faculty of Engineering and Natural Sciences, İstinye University, Istanbul 34010, Türkiye; elifnurgizem.yildirim@stu.istinye.edu.tr; 9 Department of Medical Pathology, Faculty of Medicine, İstinye University, Istanbul 34010, Türkiye; nusret.erdogan@istinye.edu.tr

**Keywords:** oleuropein, oral spray, cariogenic bacteria, antimicrobial activity, olive leaf extract, histopathological evaluation

## Abstract

**Background/Objectives**: Oleuropein is a bioactive phenolic compound from olive leaves with antimicrobial and antioxidant activity. This study aimed to develop a sprayable oral gel containing an oleuropein-rich aqueous extract and to evaluate its pharmaceutical performance antimicrobial efficacy and in ovo biological response. **Methods**: Oleuropein content was quantified using a validated chromatographic method. Polymeric systems were screened to select an optimized sprayable formulation. Physicochemical stability, dose uniformity, and antimicrobial activity against major cariogenic bacteria were evaluated. In ovo biological evaluation was conducted using the chick chorioallantoic membrane angiogenesis model together with histopathological examination of embryonic heart and liver tissues. **Results**: Oleuropein content was determined as 288.6 µg/mL in the olive leaf extract and 255.1 µg/mL in the final formulation. The optimized oral spray showed stable physicochemical properties, with pH maintained at 6.90 ± 0.02 and no relevant changes in viscosity during storage. The mean delivered dose per actuation was 0.128 ± 0.015 g, corresponding to 32.6 µg oleuropein per spray. The formulation exhibited inhibitory activity against all tested cariogenic microorganisms, with MIC values ranging from 13.3 to 170.7 µg/mL and MBC values generally two-fold higher. In the CAM assay, significant concentration- and time-dependent antiangiogenic effects were observed after 24–48 h at moderate and higher concentrations. Histopathological evaluation revealed dose-dependent acute degenerative and congestive changes in heart and liver tissues without evidence of fibrosis or steatosis. **Conclusions**: The oleuropein-based sprayable oral gel is a promising localized delivery system with adequate stability dose uniformity and antimicrobial efficacy. In ovo findings provide a conservative assessment of systemic exposure and support further development for oral biofilm and caries-related applications.

## 1. Introduction

Dental caries is a biofilm-driven disease resulting from the ecological imbalance of the oral microbiota, with specific microorganisms initiating and sustaining the demineralization process. *Streptococcus mutans* and *Streptococcus sobrinus* are primary contributors to the onset of caries due to their strong adhesion, acidogenic capacity and biofilm formation potential [1,2]. Aciduric species such as *Lactobacillus casei*, *Lactobacillus acidophilus* and *Bifidobacterium dentium* play an important role in lesion progression, particularly within deep and low-pH microenvironments [3,4].

Conventional antimicrobial agents such as chlorhexidine remain widely used in oral health; however, their long-term use is limited by adverse effects including staining, taste alteration and increased calculus accumulation [5,6]. These limitations have increased interest in natural, biocompatible compounds that can be incorporated into dental delivery systems (e.g., gels, varnishes and mucoadhesive sustained-release platforms) to reduce early colonization, acid production and biofilm maturation [7,8].

Olive leaf (*Olea europaea* L.) is a non-edible byproduct of olive cultivation and represents a rich source of bioactive secoiridoids, particularly oleuropein. Olive leaf extracts have been extensively reported to exhibit antioxidant, anti-inflammatory and antimicrobial properties [9,10]. Oleuropein, the major phenolic constituent of olive leaves, has demonstrated inhibitory activity against a broad spectrum of pathogenic microorganisms, including oral pathogens, and its antimicrobial efficacy has been shown to be enhanced by the presence of accompanying phenolic compounds [11,12]. Earlier studies reported broad-spectrum antimicrobial activity of oleuropein and related phenolics [13]. More recent findings indicate that olive leaf extracts may reduce *S. mutans* adhesion and acid production, suggesting potential to interrupt early cariogenic processes [14]. Plant-derived phenolics can also modulate quorum sensing pathways and biofilm architecture [15,16].

Oleuropein is a highly polar secoiridoid glycoside, predominantly localized in the vacuolar compartments of olive leaf cells, which makes it particularly amenable to extraction using aqueous systems. The use of water as an extraction solvent aligns with green chemistry principles, minimizing solvent toxicity, residual contamination and downstream purification burden, especially for pharmaceutical and oral–mucosal applications. Several studies have demonstrated that aqueous extraction effectively preserves oleuropein integrity while avoiding degradation associated with organic solvents and elevated temperatures. Moreover, water-based extraction has been shown to promote swelling of plant cell walls, enhance mass transfer and facilitate the release of hydrophilic phenolic compounds, including oleuropein, without introducing solvent-related safety concerns [17].

From a formulation perspective, aqueous extracts are inherently compatible with hydrophilic polymeric systems used in gels, sprays and mucoadhesive dosage forms, enabling direct incorporation without solvent exchange steps. Therefore, aqueous extraction represents a rational, safe and application-oriented approach for obtaining oleuropein-rich olive leaf extracts intended for pharmaceutical and dental formulations. Despite these promising reports, quantitative susceptibility data specifically focused on key cariogenic species remain limited. Foundational MIC and MBC data are essential before incorporation into any dental delivery system can be rationally planned. Therefore, in our study, an oral spray formulation containing an oleuropein-rich olive leaf extract was developed and evaluated against representative cariogenic bacteria to support subsequent formulation development and biocompatibility studies. The aim was to determine the MIC and MBC values of this oral spray. In addition, preliminary biocompatibility and toxicity assessments were performed using a chicken embryo model, supported by histopathological evaluation, to provide a foundational dataset for future formulation development and clinical translation. The chick chorioallantoic membrane (CAM) assay is a well-established in ovo model that allows the evaluation of angiogenic and anti-angiogenic responses in a highly vascularized, easily accessible environment [18]. Its naturally rich capillary network and short experimental timeline make it a practical alternative to mammalian models for preliminary drug screening. In this study, the anti-angiogenic potential of the tested compound was quantified using a scoring system based on vessel density in the CAM, providing a rapid and reliable assessment of vascular inhibition. The chick embryo model provides a practical and cost-effective in vivo platform to assess the biological and toxicological effects of novel compounds [19]. It allows for controlled delivery, short experimental timelines, and direct histopathological evaluation of organ-specific responses.

## 2. Materials and Methods

### 2.1. Materials

Olive leaves (*Olea europaea* L.) used in this study were collected from cultivated, non-protected olive trees in Gömeç, Balıkesir, Türkiye (Aegean region), a well-established olive-growing area. The sampling location coordinates were 39.388943° N and 26.839497° E (39°23′20.1948″ N, 26°50′22.1892″ E).

Olive leaves were obtained during routine agricultural pruning, and only small, controlled amounts were collected exclusively for academic and experimental purposes. No commercial or large-scale harvesting was performed. *Olea europaea* L. is not listed as an endangered or protected species under CITES. Oleuropein reference standard (EXTRASYNTHESE) was used for calibration.

Pharmaceutical polymers and excipients used for screening included hydroxypropyl cellulose (HPC), carboxymethyl cellulose (CMC), methyl cellulose (MC), gellan gum, carbomer, citrus pectin and gum arabic; preservatives (Easy Safe) were evaluated where applicable.

### 2.2. Conventional Extraction of Olive Leaves

Olive leaf extraction was performed using ultrapure water to eliminate potential interference from inorganic ions and to ensure reproducibility and analytical accuracy during oleuropein quantification. Instead of obtaining the highest Oleuropein content, an extraction ratio of 1–10 (leaf-water) was applied, which is suitable for oral spray content and does not contain alcohol. Conventional extraction studies were conducted on olive leaves using a similar ratio and solvent [17].

### 2.3. Instrumentation and Chromatographic Conditions

An analytical method was developed using the oleuropein reference standard. Calibration curves were plotted and basic validation checks (repeatability and robustness) were performed prior to sample measurements.

High Performance Liquid Chromatography (HPLC) analyses of the samples were carried out using a Thermo Scientific UltiMate 3000 HPLC system equipped with a dual gradient pump (DGP-3600SD), an autosampler (WPS-300TSL), a thermostatted column compartment (TCC-3000SD), and a diode array detector (DAD-3000) (Thermo Scientific, Germering, Germany) Chromatographic separation was achieved on a reversed-phase column (GL Sciences, Tokyo, Japan; Inertsil ODS-3, 150 mm × 4.6 mm i.d., 5 μm particle size). Data acquisition, system control, and chromatographic analysis were performed using Thermo Scientific™ Chromeleon™ 7.2 Chromatography Data System software (Thermo Fisher Scientific, Bremen, Germany).

**Figure 1:**
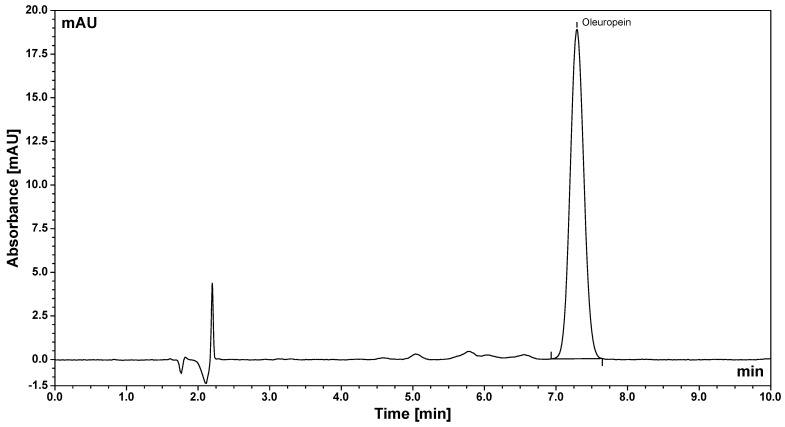
Chromatogram of oleuropein standard solution (50 µg/mL).

Oleuropein analysis was performed using an isocratic elution mode. The mobile phase consisted of acetonitrile and 0.1% (*v*/*v*) formic acid in water at a ratio of 25:75 (*v*/*v*). All mobile phases were degassed in an ultrasonic bath prior to HPLC analysis. The isocratic elution was conducted at a flow rate of 1.0 mL/min, with an injection volume of 20.0 μL. The temperatures of both the chromatographic column and the autosampler were maintained at 25 °C. Each chromatographic run was set to 10 min, followed by an additional 5 min column washing step, resulting in a total analysis time of 15 min. Oleuropein was detected using a diode array detector at a wavelength of 280 nm, with a retention time of 7.27 min. The chromatogram of the standard oleuropein solution is shown in Figure 1.

Oleuropein standard (HPLC purity ≥ 98%) was purchased from Extrasynthese (Genay, France). A stock solution of oleuropein was prepared by dissolving the standard in deionized water and stored at 4 °C until use. For calibration, a series of standard solutions with concentrations ranging from 2.5 to 180 µg/mL were prepared by serial dilution of the stock solution with deionized water. Prior to HPLC analysis, both standard and sample solutions were filtered through a 0.45 µm nylon syringe filter. To prevent column clogging and extend column lifetime, all samples were diluted fourfold before injection into the chromatographic system. Each standard and sample solution was analyzed in triplicate. The oleuropein content in the samples was quantified by external standard calibration using the oleuropein standard solutions.

### 2.4. Preparation and Screening of Candidate Formulations

A structured preliminary formulation screening was conducted to assess the suitability of an aqueous oleuropein-containing olive leaf extract for oral spray gel formulations. Pharmaceutically acceptable polymers and polymer combinations were selected based on rational criteria, including prior use in oral or mucosal formulations, compatibility with aqueous and phenolic-rich systems, ability to modulate viscosity within a sprayable range, and potential to enhance surface retention through hydration and polymer–mucosa interactions. Polymer systems representing different functional classes were evaluated individually or in binary combinations, including cellulose derivatives (hydroxypropyl cellulose, carboxymethyl cellulose, methyl cellulose), natural polysaccharides (arabic gum, citrus pectin), and ion-responsive gelling agents (gellan gum).

All formulations were prepared under identical conditions to minimize process-related variability and were screened based on viscosity behavior, gel formation tendency, visual clarity, sprayability through a standard pump system, and qualitative adhesion or retention on mucosa-like surfaces. Clarity and phase separation were assessed by visual inspection, gelation behavior was evaluated based on structural integrity and flow properties, and sprayability was assessed by observing droplet formation and dispersion upon application. Short-term physical stability observations were conducted to exclude formulations showing rapid phase separation, excessive gelation, or loss of spray performance. Based on this multi-parameter screening, a clear and sprayable formulation with moderate viscosity and improved surface retention was selected for downstream physicochemical, microbiological, and biological characterization (Table 1).

### 2.5. Physicochemical Characterization of the Optimized Formulation

The physicochemical characterization of the optimized oral spray formulation was performed to evaluate its stability, homogeneity and suitability for intraoral application during short- to mid-term storage. Particular emphasis was placed on parameters that directly influence spray performance, patient acceptability and formulation integrity, including refractive index, viscosity and pH.

Refractive index measurements were conducted as an indirect indicator of formulation homogeneity and compositional stability, allowing detection of potential phase separation, polymer rearrangement or solute precipitation over time. Measurements were carried out using a calibrated digital refractometer at a controlled temperature (24.3 °C), as refractive index values are sensitive to temperature fluctuations. Repeated measurements were performed at predefined storage intervals over a three-month period.

Viscosity measurements were performed using a vibro-viscometer (SV-10, A&D Company, Tokyo, Japan), operating based on the tuning-fork vibration principle. The instrument measures viscosity by monitoring the damping of vibration of two sensor plates oscillating at a constant frequency (30 Hz) when immersed in the formulation, allowing viscosity determination without applying rotational shear. This approach is particularly suitable for polymer-containing, semi-viscous aqueous systems such as sprayable oral gels [20]. All measurements were conducted at a controlled temperature of 24.3 ± 0.5 °C after equilibration of samples for at least 15 min. Viscosity measurements were performed in triplicate (*n* = 3) at each predefined storage time point (months 0–3), and results were expressed as mean ± standard deviation. Viscosity values were reported in relative units, as the primary objective was comparative stability monitoring over time rather than full rheological characterization.

The pH of the formulation was measured to assess chemical stability and compatibility with the oral environment. pH monitoring is particularly relevant for formulations intended for intraoral use, as deviations from near-neutral pH may lead to mucosal irritation, instability of phenolic compounds or degradation of polymeric excipients. pH measurements were performed using a calibrated pH meter, and values were recorded at each time point during the storage period. All measurements were conducted in triplicate, and the mean values were used to evaluate stability trends.

### 2.6. Determination of Dose Uniformity

The oral spray formulation was filled into identical spray containers equipped with the same pump mechanism. Prior to measurement, each container was equilibrated at room temperature (24 ± 1 °C) to minimize variability due to temperature-dependent viscosity changes. The spray device was primed by actuating the pump three times, and these initial actuations were discarded to ensure consistent spray performance.

For dose determination, the container was weighed using a calibrated analytical balance (precision ±0.1 mg) immediately before and after twenty consecutive spray actuations. Sprays were applied vertically into the air at a constant distance to avoid material loss due to splashing or condensation. The total weight loss corresponding to twenty actuations was recorded, and the average delivered mass per single actuation was calculated by dividing the cumulative weight loss by twenty.

This procedure was repeated for multiple consecutive measurement points to evaluate intra-device dose consistency and delivered dose uniformity. The mean delivered dose per actuation, standard deviation (SD), and relative standard deviation (RSD, %) were calculated as indicators of dose uniformity. The calculated dose per spray was subsequently used to define the nominal dose of oleuropein administered per actuation, taking into account the measured oleuropein concentration in the formulation.

### 2.7. Microorganisms and Culture Conditions

Reference strains were obtained from the American Type Culture Collection (ATCC) and confirmed by matrix-assisted laser desorption/ionization time-of-flight mass spectrometry (MALDI-TOF MS; VITEK^®^ MS system, BioMérieux, France). The bacterial species and corresponding ATCC numbers are listed in Table 2.

*Streptococcus mutans* and *Streptococcus sobrinus* were cultured in Mueller–Hinton broth supplemented with 5% lysed horse blood to support streptococcal growth, in accordance with European Committee on Antimicrobial Susceptibility Testing (EUCAST) antimicrobial susceptibility testing guidelines for fastidious organisms. *Lactobacillus casei* and *Lactobacillus acidophilus* were grown in de Man, Rogosa and Sharpe (MRS) broth under aerobic conditions, while *Bifidobacterium dentium* was cultured in MRS broth supplemented with 0.05% (*w*/*v*) L-cysteine hydrochloride under anaerobic conditions.

Anaerobiosis for *B. dentium* was achieved using an anaerobic jar system with gas-generating sachets producing an atmosphere of H_2_/CO_2_/N_2_. All bacterial cultures were incubated at 37 °C, and inocula were prepared from freshly grown cultures adjusted to a 0.5 McFarland standard (approximately 1.5 × 10^8^ CFU/mL) prior to antimicrobial susceptibility testing.

### 2.8. Determination of Minimum Inhibitory Concentration (MIC)

The minimum inhibitory concentration (MIC) of oleuropein was determined using the broth microdilution method in sterile 96-well plates, in accordance with the EUCAST guidelines for fastidious microorganisms. Oleuropein was serially two-fold diluted in Mueller–Hinton broth supplemented according to the growth requirements of each bacterial species, to obtain final concentrations ranging from 1 to 512 µg/mL.

Each well was inoculated with a standardized bacterial suspension adjusted to approximately 5 × 10^5^ CFU/mL, resulting in a final assay volume of 200 µL per well. Sterility controls (medium only) and growth controls (medium plus inoculum without oleuropein) were included in each plate. Chlorhexidine was used as a reference antimicrobial for *Streptococcus* species, while ampicillin served as the reference agent for *Lactobacillus* species and *Bifidobacterium dentium*, using concentration ranges recommended by EUCAST.

Plates were incubated at 37 °C for 24 h under 5% CO_2_ for facultative anaerobic species, with strict anaerobic conditions were applied for *B. dentium* using an anaerobic jar system with gas-generating sachets. The MIC was defined as the lowest concentration of oleuropein that resulted in no visible bacterial growth compared with the growth control. Growth inhibition was further confirmed by measuring optical density at 600 nm (OD_600_), and MIC values were defined as the lowest concentration showing no detectable increase in OD_600_ compared with the growth control.

### 2.9. Determination of Minimum Bactericidal Concentration (MBC)

The minimum bactericidal concentration (MBC) was determined by subculturing 10 µL aliquots from wells showing no visible growth in the MIC assay onto Mueller–Hinton agar plates supplemented as required for each microorganism. For streptococcal species, supplemented agar media appropriate for fastidious organisms were used, while incubation conditions were adjusted according to the growth requirements of each bacterial species. Plates were incubated at 37 °C for 24 h under aerobic, CO_2_-enriched, or anaerobic conditions, as appropriate.

The MBC was defined as the lowest concentration of oleuropein that resulted in no detectable colony growth on agar plates, corresponding to a ≥99.9% reduction in the initial bacterial inoculum. All experiments were performed in three independent biological experiments, each conducted in technical triplicate, and results are presented as the mean ± standard deviation (SD).

### 2.10. Angiogenesis Studies for Oral Spray Formulation

The effects of oral spray formulation on angiogenesis were examined using the chick chorioallantoic membrane (CAM) assay. The eggs were gently cleaned with a tissue paper moistened with 70% ethanol, and the air sacs were marked under light before being placed in an incubator. The incubation conditions were maintained at 60–70% humidity and 37 °C [21].

The day of arrival at the laboratory was considered egg development day (EDD) 0. On EDD0, a small hole was drilled above the air sac, and an additional hole was made on the lateral side of the egg to release air from the air sac and allow air entry into the egg. The eggs were then incubated until EDD3. On EDD3, approximately 3 mL of albumin was withdrawn through the lateral hole using a sterile syringe to facilitate the detachment and downward movement of the CAM, thereby creating space for the subsequent assay. The eggs were further incubated until EDD5 [22]. On EDD5, a circular window (~2 cm in diameter) was carefully opened on the upper shell under sterile conditions. Oral spray formulation was applied onto the CAM surface at logarithmic dilution doses (1:1–1:1000) prepared in PBS, in a final volume of 50 µL. The CAM surface was photographed at the time of application and once daily for the following two days to monitor vascular changes. Images were evaluated according to the predefined scoring criteria (Table 3) [23]. Scoring was performed independently by two blinded observers who were unaware of the treatment groups to ensure objectivity and reproducibility. Statistical analysis was performed using one-way ANOVA followed by Tukey’s post hoc test (Equation (1)). In ovo anti-angiogenic scoring system.

Equation (1) Calculation of the average anti-angiogenic score in the CAM assay

(1) $$Average\;score=\frac{\left(Score\;2\right)\;\#egg\times 2+\left(Score\;1\right)\;\#egg}{Total\;\#egg\;scored\;}$$

All in ovo experiments were performed on fertilized chicken eggs before embryonic day 14. The incubation period of chicken embryos is 21 days, and animal experimentation regulations apply only to the last third of this period. Accordingly, experiments conducted before embryonic day 14 are not classified as animal experiments and do not require approval from an animal ethics committee, in accordance with Directive 2010/63/EU.

### 2.11. Histopathological Evaluation of Oral Spray Formulation

Fertilized ROSS 308 chicken eggs (Pak Tavuk, Türkiye) were incubated at 37 °C and 60–70% relative humidity under constant rotation until embryonic development day 5 (EDD5). At this stage, oral spray formulation was administered intravenously (i.v.) into the chorioallantoic vein at four different concentrations: 1:1000 (*v*/*v*), 1:100 (*v*/*v*), 1:1 (*v*/*v*), and neat (100%, *v*/*v*). A separate control embryo received phosphate-buffered saline (PBS). Each concentration group included one embryo. After 48 h of exposure, embryos were sacrificed, and heart and liver tissues were carefully excised for histopathological evaluation. The tissues were fixed in 10% neutral-buffered formalin (NBF), routinely processed, embedded in paraffin, sectioned at 5 µm, and stained with hematoxylin and eosin (H&E) using standard histological procedures [24].

## 3. Results

### 3.1. Extracted Olive Leaves

A controlled 1–10 ratio allows effective hydration and swelling of the olive leaf matrix, facilitating solvent penetration into intracellular compartments where oleuropein is concentrated. Ultrapure water prevents catalytic degradation or complexation reactions that may occur in the presence of metal ions commonly found in tap or deionized water. The solid-to-liquid ratio was selected to balance extraction efficiency, diffusion kinetics and practical scalability. Previous studies have shown that moderate solid-to-liquid ratios in aqueous systems are sufficient to achieve high oleuropein recovery while avoiding excessive dilution and prolonged extraction times [25]. Additionally, maintaining a defined olive leaf–water ratio ensures batch-to-batch consistency and enables direct comparison with literature data on aqueous olive leaf extracts. This approach supports both analytical robustness and downstream formulation compatibility, particularly for gel and spray-based delivery systems where solvent composition and concentration play a critical role in physicochemical stability and performance.

### 3.2. Quantification of Oleuropein in the Samples

The linearity of the method was evaluated by analyzing seven different concentrations of the corresponding aqueous standard solutions, selected based on their relative abundance in olive leaf samples. Calibration curves were constructed by plotting the detector response against the analyte concentration (µg/mL) in the aqueous standard solutions, within the appropriate concentration range for each compound.

Under the chromatographic conditions applied in this study, the calibration curve exhibited excellent linearity, as confirmed by regression analysis (R^2^ > 0.999). The regression equation was determined as *y* = 0.0824*x* + 0.0694. Oleuropein showed linear detector response over the concentration range of 2.5–180 µg/mL. The linearity of the calibration curve was further verified by both the correlation coefficient and visual inspection of the calibration plot.

The limits of detection (LOD) and quantification (LOQ) were determined as 0.14 µg/mL and 0.43 µg/mL, respectively, corresponding to the lowest standard concentration exhibiting a relative standard deviation (%RSD) ≤ 2%. The LOD and LOQ values were calculated using the equations LOD = 3.3 × SD and LOQ = 10 × SD, where SD represents the standard deviation of the peak areas (*n* = 6) obtained from replicate analyses of the lowest concentration level of the standard solution.

**Figure 2:**
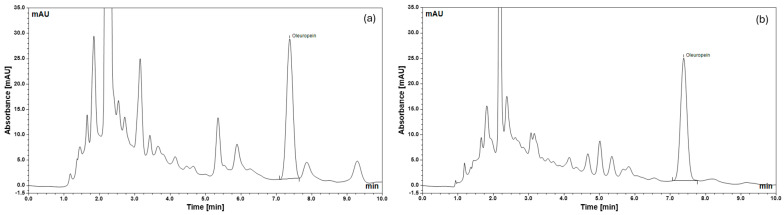
The chromatograms of the extract of olive leaves (**a**) and oral spray (**b**).

The chromatographic profiles of oleuropein present in the olive leaf extract and the formulated sample are shown in Figure 2. The oleuropein contents of the olive leaf extract and the oral spray were determined to be 288.6 µg/mL and 255.1 µg/mL, respectively.

### 3.3. Formulation Screening and Selection

Across the screened excipients and combinations, clear and sprayable candidates were obtained primarily with cellulose derivatives and Arabic gum-based blends. Formulations combining 0.5% CMC with 1% Arabic gum showed favorable clarity, gelation and retention characteristics, while remaining sprayable; HPC–Arabic gum combinations were also sprayable but generally had lower retention. These screening outcomes informed selection of an optimized sprayable and mucoadhesive candidate for characterization (Table 4).

### 3.4. Characterization Data of Optimized Formulation

The physicochemical parameters evaluated in this study were assessed using functionally defined, application-oriented criteria appropriate for an early-stage formulation feasibility and stability assessment rather than fixed pharmacopeial specifications. Refractive index, viscosity, and pH were selected as formulation-relevant indicators to support interpretation of physical stability and suitability for oral spray application.

Refractive index was monitored as an indirect indicator of formulation homogeneity and compositional stability. In aqueous polymer-based systems, consistency of refractive index values over time suggests the absence of phase separation, polymer precipitation, or solute redistribution. Accordingly, the consistently observed refractive index of approximately 2.9, with minimal variation over the three-month storage period, was interpreted as indicative of a physically homogeneous and stable formulation and was used as a comparative stability marker rather than an absolute acceptance limit.

Viscosity was evaluated to ensure compatibility with both sprayability and surface retention. The measured viscosity of approximately 1.6 relative units, determined by vibroviscometry, reflects a moderate consistency suitable for pump-based oral spray gels. In this screening-level study, viscosity criteria were defined functionally: values were required to permit reproducible spray actuation and droplet formation while remaining sufficiently high to limit rapid runoff from moist oral surfaces. The absence of significant viscosity changes over time was interpreted as evidence of polymer network stability and resistance to over-gelation or structural degradation during storage.

The pH of the optimized formulation was monitored as a critical parameter for chemical stability, polymer compatibility, and oral mucosal tolerance. A near-neutral pH of 6.9 was selected to minimize the risk of mucosal irritation, preserve the stability of oleuropein and other phenolic constituents, and maintain the integrity of the polymeric matrix. The narrow pH variation observed throughout the storage period was therefore considered supportive of both chemical and physical stability.

Overall, stability of these physicochemical parameters over time, rather than their absolute numerical values (Table 5), was used as the primary indicator of formulation robustness and suitability for oral spray application at this stage of development.

### 3.5. Results of Delivered Dose Uniformity

The delivered dose of the oral spray formulation was evaluated using a gravimetric approach in order to assess dose reproducibility and content uniformity under repeated actuation conditions. The cumulative weight loss measured after 20 consecutive spray actuations showed a high level of consistency, indicating stable pump performance and uniform dose delivery.

Delivered dose uniformity was evaluated gravimetrically based on 20 consecutive spray actuations. Mean delivered dose, standard deviation (SD), and relative standard deviation (RSD) were calculated to assess dose reproducibility of the oral spray formulation.

As summarized in Table 6, the total weight loss recorded after 20 spray actuations exhibited minimal variability between repeated measurements. When normalized per actuation, the calculated mean delivered dose per spray showed low standard deviation, demonstrating that each actuation delivered a comparable amount of formulation. This finding confirms that the spray system provides reliable and reproducible dosing, which is critical for oral applications where precise administration of active compounds is required.

Importantly, no progressive decrease or increase in delivered mass was observed across the twenty consecutive sprays, suggesting the absence of pump fatigue, clogging, or formulation-related flow instability during repeated use. The uniformity of the delivered dose further supports the homogeneity of the formulation and its suitability for controlled administration.

Based on these results, content uniformity was achieved at each spray actuation, and the oral spray system met the practical requirements for consistent dose delivery. The gravimetrically determined mean dose per actuation was subsequently used to calculate the nominal amount of oleuropein delivered per spray, ensuring accurate interpretation of antimicrobial and formulation performance outcomes reported in this study.

The relative standard deviation (RSD) of 11.7% observed for the delivered dose per actuation is considered acceptable for a viscous, polymer-containing oral spray formulation. Previous studies and regulatory guidelines indicate that higher variability is expected for mucoadhesive and viscosity-modified liquid sprays compared to solid dosage forms, provided that no systematic dose drift occurs. The consistent delivered dose observed across consecutive actuations confirms the robustness of the spray system and formulation homogeneity.

### 3.6. Antimicrobial Activity (MIC and MBC) of Olive Leaf Extract-Based Oral Spray Formulation

The oral spray formulation inhibited all tested strains, with MIC values ranging from approximately 13 to 171 µg/mL. MBC values were generally two-fold higher than the corresponding MIC values, consistent with a concentration-dependent bactericidal activity. Reference antimicrobials displayed consistently lower MIC and MBC values, thereby confirming the validity and reliability of the assay conditions (Table 7).

### 3.7. Angiogenesis Results for Olive Leaf Extract Based Oral Spray Formulation

**Figure 3:**
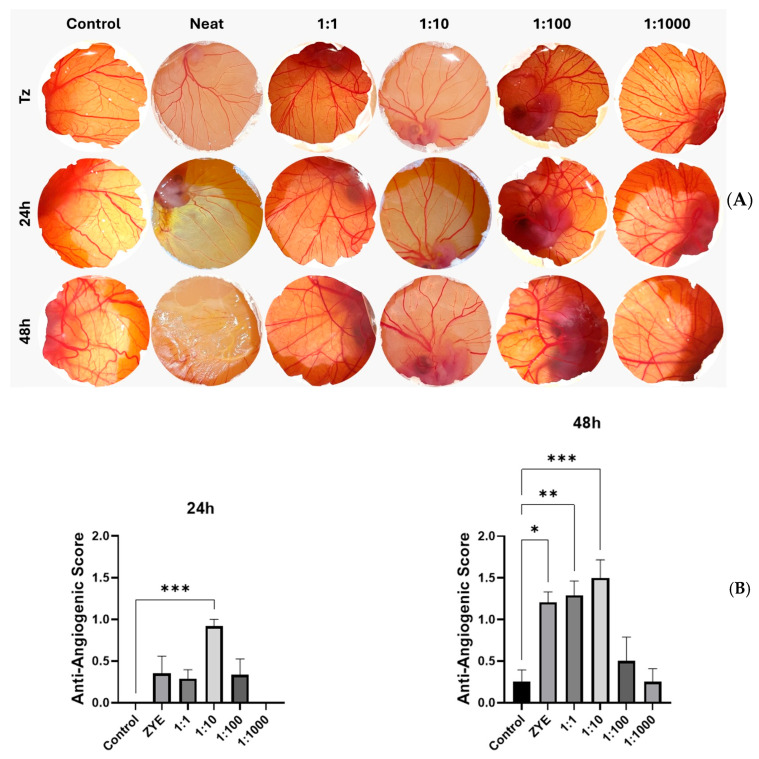
Anti-angiogenic effects of formulation on the chick chorioallantoic membrane (CAM) at 24 h and 48 h. (**A**) Representative CAM images for 24 h and 48 h following treatment with Oral spray at various dilution ratios (1:1, 1:10, 1:100, 1:1000). Quantitative angiogenesis scores of the CAM assay at 24 h and 48 h following treatment with formulation and its serial dilutions (1:1, 1:10, 1:100, 1:1000). *: *p* < 0.05, **: *p* < 0.01, and ***: *p* < 0.001. Data are presented as mean ± SD (**B**).

Quantitative evaluation of the CAM images revealed a time- and dose-dependent anti-angiogenic response to oral spray. At 24 h, a statistically significant inhibition of angiogenesis was observed only in the 1:10 dilution group (*p* < 0.05), which exhibited an average anti-angiogenic score of approximately 1, corresponding to a strong reduction in vessel area. The undiluted formulation and 1:1 group showed a tendency toward inhibition, though not statistically significant at this time point. At 48 h, the inhibitory effects became more pronounced and extended to higher concentrations. The undiluted formulation, 1:1, and 1:10 dilutions demonstrated statistically significant reductions in vascularization, with *p* < 0.05, *p* < 0.01, and *p* < 0.001, respectively. The mean anti-angiogenic scores for these groups ranged between 1 and 1.5, reflecting a marked suppression of vessel formation and the presence of partially avascular zones on the CAM surface. Lower dilutions (1:100 and 1:1000) did not differ significantly from controls (Figure 3). These findings indicate that X exerts a potent and sustained anti-angiogenic effect on the CAM, particularly at moderate to high concentrations.

### 3.8. Histopathological Evaluation Results of Oral Spray Formulation

Microscopic examination of heart and liver tissues revealed dose-dependent histopathological alterations following intravenous administration of oral spray formulation.

**Figure 4:**
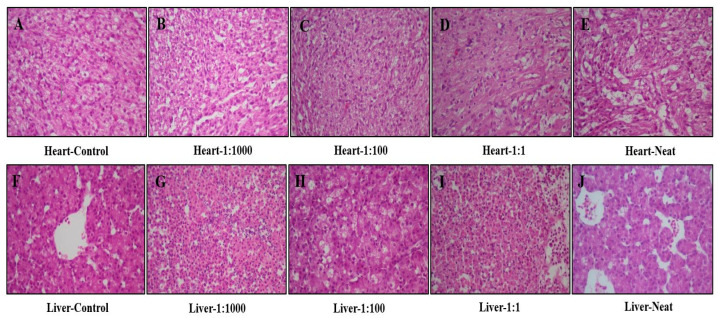
Representative H&E-stained sections of heart (**A**–**E**) and liver (**F**–**J**) tissues from chick embryos following intravenous administration of oral spray at increasing concentrations (*v*/*v*). Panels (**A**,**F**) (Control) show preserved myocardial and hepatic architecture. Panels (**B**,**G**) (1:1000, *v*/*v*) display moderate myocyte/hepatocyte degeneration with vascular or sinusoidal congestion. Panels (**C**,**H**) (1:100, *v*/*v*) show mild degeneration with focal necrosis/apoptosis. Panels (**D**,**I**) (1:1, *v*/*v*) demonstrate interstitial edema and inflammatory cell infiltration. Panels (**E**,**J**) (Neat, 100%, *v*/*v*) exhibit pronounced degeneration, necrosis/apoptosis, and congestion. Hematoxylin and eosin (H&E), 40× magnification.

Heart Tissue: The control group exhibited normal myocardial architecture, characterized by regularly aligned muscle fibers and centrally located nuclei. No evidence of degeneration, necrosis/apoptosis, or inflammatory changes was detected, while only mild interstitial edema was observed in some regions. In the 1:1000 (*v*/*v*) group, moderate myocyte degeneration and marked vascular congestion were evident. The 1:100 (*v*/*v*) group showed mild myocyte degeneration, focal necrosis/apoptosis, and slight interstitial edema with mild vascular congestion. The 1:1 (*v*/*v*) group demonstrated increased interstitial edema and inflammatory cell infiltration accompanied by mild myocyte degeneration and necrosis/apoptosis. In the neat (100%, *v*/*v*) group, moderate myocyte degeneration and vascular congestion were observed together with mild interstitial edema and inflammatory infiltration. No fibrotic alterations were detected in any of the groups. Representative photomicrographs of the cardiac tissue sections are shown in Figure 4 (H&E, 40× magnification).

Liver Tissue: Histopathological examination of liver sections revealed progressive hepatocellular injury with increasing formulation concentrations. The control group displayed normal lobular organization and preserved hepatocyte morphology without evidence of degeneration or inflammation. At 1:1000 (*v*/*v*), mild hepatocyte degeneration and sinusoidal congestion were detected. The 1:100 (*v*/*v*) group exhibited moderate hepatocellular degeneration with mild necrosis/apoptosis and mild congestion. In the 1:1 (*v*/*v*) group, moderate-to-severe hepatocyte degeneration, necrosis/apoptosis, and sinusoidal congestion were accompanied by mild inflammatory infiltration. The neat (100%, *v*/*v*) group demonstrated extensive hepatocellular degeneration, focal necrosis/apoptosis, and marked sinusoidal congestion with mild inflammatory infiltration. No steatosis or fibrosis was observed in any group. Representative photomicrographs of hepatic sections are presented in Figure 4 (H&E, 40× magnification).

Microscopic examination was performed under a light microscope at 40× magnification. Histopathological parameters—including cellular degeneration, necrosis/apoptosis, inflammatory infiltration, vascular congestion, interstitial edema, steatosis, and fibrosis—were examined descriptively and semi-quantitatively by an independent pathologist The severity of observed alterations was graded as “-” (no change), “+” (mild), “++” (moderate), and “+++” (severe) according to the criteria summarized in Table 8 and Table 9.

## 4. Discussion

In the present study, the biological activity of olive leaf extract was evaluated using a standardized formulation based on oleuropein content, which is widely recognized as the predominant phenolic compound in olive leaves and the principal bioactive constituent of olive leaf extract (OLE). Oleuropein typically constitutes the largest fraction of olive leaf polyphenols, accounting for approximately 6 to 9% of the dry leaf weight, which is substantially higher than the levels of other individual phenolics such as hydroxytyrosol or verbascoside [26]. Accordingly, commercial OLE products are commonly standardized by oleuropein content (often ~15–20% *w*/*w* oleuropein) to ensure batch-to-batch consistency [27]. This practice reflects the compound’s status as the principal marker for quality and potency in OLE formulations.

In scientific literature, oleuropein is frequently cited as the main contributor to olive leaf’s biological activities. As the dominant phenolic, it has been linked to a broad spectrum of health effects and antimicrobial properties attributed to OLE. In fact, oleuropein has been described as “the main phenolic component” of the olive plant, responsible for many of the extract’s antioxidant, anti-inflammatory, cardioprotective, and other beneficial actions [28]. These findings underscore a consensus that oleuropein serves as the primary active ingredient in olive leaf preparations, validating its use as the key reference compound in standardization and in discussions of OLE’s bioactivity.

While oleuropein is the principal quantified component, the HPLC chromatogram also revealed the presence of additional phenolic compounds, which may contribute to the observed biological effects. Minor constituents such as hydroxytyrosol, tyrosol, verbascoside, and luteolin derivatives have been reported to exhibit antimicrobial, anti-inflammatory, and antibiofilm activity [29,30,31]. While these co-extracted compounds were not individually quantified or assessed in this study, their potential synergistic or additive contributions cannot be excluded. This limitation has been acknowledged, and future studies involving fractionation or mechanistic profiling could provide a more detailed understanding of the relative roles of individual components in olive leaf-based formulations.

Although no quantitative mucoadhesive test was performed in the present study, the observed retention behavior of selected formulations can be rationally discussed based on polymer characteristics and qualitative surface interactions. Cellulose derivatives, particularly carboxymethylcellulose (CMC), are well known for their ability to hydrate, swell and form hydrogen bonds with mucin glycoproteins, contributing to prolonged surface residence in oral and mucosal applications. Arabic gum, on the other hand, provides a structured gel network that enhances cohesion and limits rapid runoff from moist surfaces.

The combination of CMC and arabic gum resulted in a formulation that exhibited visible surface retention while remaining sprayable, suggesting a favorable balance between fluidity and adhesive interaction. In the context of dental and oral delivery systems, such retention is considered functionally relevant, as increased residence time may enhance local exposure of antimicrobial agents to the biofilm environment without requiring strong chemical adhesion. Similar qualitative assessments of retention and residence behavior have been reported as acceptable preliminary indicators during early-stage formulation screening, prior to detailed mucoadhesion or residence-time measurements.

To ensure accurate and reproducible dosing during oral spray administration, the delivered dose per actuation was determined by a gravimetric method based on cumulative weight loss measurements. This approach is widely used for spray-based pharmaceutical and dental formulations, as it provides a simple and reliable estimation of the actual amount dispensed per spray.

From a formulation science perspective, the physicochemical characterization in this study was intentionally conducted at a screening and feasibility level. Viscosity measurements were primarily used to monitor formulation stability and reproducibility over time rather than to establish a complete rheological profile. Therefore, advanced rheological analyses such as shear-rate dependent viscosity, viscoelastic parameters, or yield stress determination were not included. In addition, quantitative mucoadhesion testing and spray performance analyses, including plume geometry and droplet size distribution, were beyond the scope of the present work. These parameters are recognized as important for advanced formulation optimization and will be addressed in future studies. Accordingly, the current findings should be interpreted as demonstrating formulation feasibility and short-term stability rather than final optimization.

This study provides quantitative MIC and MBC data for oleuropein against representative cariogenic bacteria, addressing a practical gap needed to guide dental formulation development. Oleuropein showed measurable activity across *Streptococcus*, *Lactobacillus* and *Bifidobacterium* species, with bactericidal concentrations generally one- to two- fold higher than the inhibitory concentrations. These findings are consistent with prior reports of broad-spectrum antimicrobial and antibiofilm effects of oleuropein and related olive phenolics [13,32].

While chlorhexidine remains a benchmark agent for caries-related biofilm control, adverse effects limit long-term use [5,6,33]. Natural antimicrobials such as oleuropein may offer a complementary approach, particularly if incorporated into localized delivery platforms that support retention and sustained exposure. The formulation screening conducted in this study indicates that polymer selection can balance clarity, sprayability and mucoadhesive retention, supporting feasibility for intraoral delivery.

Limitations include the in vitro nature of the assays, use of a limited set of reference strains and lack of cytocompatibility testing on oral tissues. Future work should evaluate mechanisms (e.g., effects on adhesion, acid production and EPS synthesis), biocompatibility on oral epithelial and fibroblast models, and antibiofilm efficacy in polymicrobial settings and in vivo models [34,35].

The chorioallantoic membrane (CAM) model has been widely used not only for angiogenesis and tumor progression studies but also as a preclinical in vivo platform for evaluating pharmacological effects, biodistribution, and acute toxicity of drug formulations, serving as an intermediary step before mammalian testing [36]. Within this context, although the formulation was designed for local intraoral application, biological safety was intentionally evaluated using a systemic exposure model to obtain a conservative safety profile. Intravenous administration in the chick embryo model does not represent the intended clinical route and is therefore considered a limitation of the study; however, it was deliberately selected to assess potential off-target effects under worst-case systemic exposure conditions. This approach is relevant for intraoral formulations, as limited systemic exposure may still occur through mucosal absorption, accidental swallowing, or compromised epithelial barriers.

Within this framework, the cardiac and hepatic histopathological findings observed in the present study should be interpreted as part of an upper safety boundary rather than a direct representation of clinical intraoral application. The chick embryo model allows organ level histopathological assessment following in ovo or vascular associated administration and has been repeatedly used to detect dose dependent systemic effects under controlled conditions [37,38]. Therefore, the absence of severe or progressive tissue damage under these exaggerated exposure conditions supports a favorable safety margin for local intraoral use. Nevertheless, conclusions regarding systemic toxicity remain limited by the experimental design and should be interpreted cautiously in the context of the intended local application.

The present study demonstrated that intravenous administration of olive leaf-based oral spray formulation induced dose-dependent histopathological alterations in both cardiac and hepatic tissues of chick embryos. Although the compound was not expected to cause marked tissue injury, higher concentrations resulted in degenerative, inflammatory, and vascular changes, particularly in the 1:1 (*v*/*v*) and neat (100%, *v*/*v*) groups. The observed myocyte and hepatocyte degeneration, vascular congestion, and interstitial edema suggest that oral spray may interfere with cellular membrane stability or microcirculatory homeostasis at elevated doses. Such alterations are commonly associated with increased oxidative stress, metabolic imbalance, or transient inflammatory activation, which may contribute to the structural disorganization detected in both organs. Importantly, the absence of fibrosis or steatosis in any of the examined groups indicates that the damage was acute and non-progressive, rather than chronic. The mild to moderate nature of the lesions at intermediate doses further suggests a threshold-dependent effect, where lower concentrations may be tolerated without significant cellular disruption. Taken together, these findings indicate that oral spray formulation can induce concentration-dependent cytotoxic and degenerative responses in developing tissues. In line with the present findings, thermoresponsive sol–gel platforms based on Poloxamer 407 have previously been reported to improve local retention, controlled drug release, and patient comfort in sensitive tissue applications. However, such systems rely on temperature-dependent gelation behavior and are therefore primarily suited for formulations designed to undergo in situ gelation, whereas the present study focused on non-thermoresponsive polymer systems to ensure reproducible sprayability and controlled residence under room and oral temperature conditions [20,39,40]. Further studies including larger sample sizes, biochemical analyses, and molecular assays for oxidative and apoptotic markers are required to elucidate the mechanisms underlying these histopathological effects and to determine safe exposure levels.

## 5. Conclusions

This study demonstrates that oleuropein-rich olive leaf extract can be successfully formulated into a stable and reproducible oral spray suitable for intraoral applications. The optimized formulation showed consistent physicochemical stability, acceptable dose uniformity, and effective spray performance over the evaluated storage period.

The oral spray exhibited measurable inhibitory and bactericidal activity against key cariogenic microorganisms, confirming oleuropein as the primary contributor to the antimicrobial effect of the formulation. These findings support the use of olive leaf-derived oleuropein as a natural, locally acting agent for oral health applications, particularly in strategies aimed at reducing cariogenic bacterial load.

Preliminary in ovo angiogenesis and histopathological evaluations indicated concentration-dependent biological responses, highlighting the importance of controlled local dosing rather than systemic exposure. Overall, the results provide a solid formulation and biological foundation for further development of oleuropein-based oral spray systems targeting caries prevention and oral biofilm control.

## Figures and Tables

**Table 1:** Oral spray formulation studies.

Formulation Code	Polymer Composition (*w*/*w* %)	Olive Leaf Extract (*w*/*w*)
F1	Carbomer 1.0	99
F2	HPC 1.0	99
F3	Gellan gum 1.0	99
F4	Arabic gum 0.5 + HPC 0.5	99
F5	Arabic gum 1.0 + CMC 1.0	98
F6	CMC 1.0	99
F7	MC 0.5	99.5
F8	HPC 2.0 + Arabic gum 0.5	97.5
F9	HPC 0.5 + Gellan gum 1.0	98.5
F10	CMC 0.5 + Arabic gum 1.0	98.5
F11	MC 0.75 + Gellan gum 1.0	98.25
F12	Citrus pectin 1.0	99
F13	Citrus pectin 1.5	98.5
F14	Arabic gum 0.5 + HPC 0.5 + 1% easy safe	98

**Table 2:** Selected bacterial species and corresponding ATCC reference numbers.

Bacterial Species	American Type Culture Collection (ATCC) No
*Streptococcus mutans*	25175
*Streptococcus sobrinus*	33478
*Lactobacillus casei*	334
*Lactobacillus acidophilus*	4356
*Bifidobacterium dentium*	27678

**Table 3:** Semi-quantitative scoring system for evaluating the anti-angiogenic effect in the CAM assay.

Score	Anti-Angiogenic Effect	After Treatment Effect on CAM
0	Inactive	No change
0.5	Weak	No capillary free area
1	Strong	Small capillary free area or area with significantly reduced density of capillaries.
2	Very strong	Capillary free area around the pellet at least double the size of the pellet

**Table 4:** Evaluation of oral spray formulations.

Code	Polymer Composition (*w*/*w* %)	Olive Leaf Extract (*w*/*w* %)	Appearance	Gel/Viscosity	Sprayability	Retention	Interpretation
F1	Carbomer 1.0	99	Clear	Very high (rigid gel)	No	High	Too viscous, not sprayable
F2	HPC 1.0	99	Clear	Low	Excellent	Low	Sprayable but droplets are sliding
F3	Gellan gum 1.0	99	Clear	Strong gel	Poor	High	Limited sprayability
F4	Arabic gum 0.5 + HPC 0.5	99	Clear	Moderate gel	Moderate	Moderate	Sprayable, droplets can remain on surface
F5	Arabic gum 1.0 + CMC 1.0	98	Clear	Balanced gel	Good	High	Optimal balance, selected formulation
F6	CMC 1.0	99	Slightly opalescent	Moderate	Good	Moderate	Insufficient gel integrity
F7	MC 0.5	99.5	Clear	Low	Good	Low	Rapid runoff observed
F8	HPC 2.0 + Arabic gum 0.5	97.5	Clear	Very strong gel	No	High	Over-gelation
F9	HPC 0.5 + Gellan gum 1.0	98.5	Clear	Strong gel	Poor	High	Sprayability insufficient
F10	CMC 0.5 + Arabic gum 1.0	98.5	Clear	Balanced gel	Good	High	Comparable to F5, lower CMC
F11	MC 0.75 + Gellan gum 1.0	98.25	Slightly opaque	Rigid gel	No	High	Not sprayable
F12	Citrus pectin 1.0	99	Opaque	Irregular gel	Moderate	Moderate	Spray issues due to pectin
F13	Citrus pectin 1.5	98.5	Clear	Moderate gel	Good	Moderate–Good	Sprayable but droplets are sliding
F14	Gellan gum 0.5 + HPC 0.5 + 1 Easy Safe	98	Clear	Moderate gel	Moderate	Moderate	Sprayable, droplets can remain on surface

**Table 5:** Physicochemical Characterization of the Optimized Formulation (Mean ± SD).

Parameter	Month 0	Month 1	Month 2	Month 3
Refractive index	2.90 ± 0.00	2.90 ± 0.01	2.90 ± 0.01	2.90 ± 0.00
Viscosity	1.60 ± 0.03	1.59 ± 0.04	1.61 ± 0.03	1.60 ± 0.02
pH	6.90 ± 0.02	6.91 ± 0.03	6.89 ± 0.02	6.90 ± 0.02

**Table 6:** Content uniformity of the doses.

Parameter	Value
Number of spray actuations (*n*)	20
Mean delivered dose per actuation (g) ± SD	0.128 ± 0.015
Relative standard deviation (RSD, %)	11.7
Dose of Oleuropein per actuation (µg)	32.6

**Table 7:** MIC and MBC values (µg/mL) of oleuropein and reference antibiotics.

Bacterial Strain	Oral Spray MIC (μg/mL)	Oral Spray MBC (μg/mL)	Reference Antibiotic	Reference MIC (μg/mL)	Reference MBC (μg/mL)
*Streptococcus mutans* ATCC 25175	13.33 ± 4.62	26.67 ± 18.47	Chlorhexidine	1.0 ± 0.0	2.0 ± 0.0
*Streptococcus sobrinus* ATCC 33478	13.33 ± 4.62	26.67 ± 18.47	Chlorhexidine	1.0 ± 0.0	2.0 ± 0.0
*Lactobacillus casei* ATCC 334	21.33 ± 9.24	42.67 ± 18.47	Ampicillin	1.0 ± 0.0	2.0 ± 0.0
*Lactobacillus acidophilus* ATCC 4356	64 ± 0.0	128.0 ± 0.0	Ampicillin	1.0 ± 0.0	2.0 ± 0.0
*Bifidobacterium dentium* ATCC 27678	170.67 ± 73.93	341.33 ± 147.75	Ampicillin	2.0 ± 0.0	4.0 ± 0.0

**Table 8:** Evaluation of histopathological alterations in heart tissue following intravenous administration of oral spray at different concentrations (*v*/*v*). The severity of myocyte degeneration, necrosis/apoptosis, interstitial edema, inflammatory infiltration, and vascular congestion increased in a dose-dependent manner. Scores were graded as “-” (no change), “+” (mild), “++” (moder-ate).

Histopathological Change	Control	1:1000 (*v*/*v*)	1:100 (*v*/*v*)	1:1 (*v*/*v*)	Neat (100%, *v*/*v*)
Myocyte degeneration	-	++	+	+	++
Necrosis/apoptosis	-	-	+	+	+
Interstitial edema	-	+	+	++	+
Inflammatory infiltration	-	-	-	++	+
Vascular congestion	+	++	+	+	++
Fibrosis	-	-	-	-	-

**Table 9:** Semi-quantitative evaluation of histopathological alterations in liver tissue following intravenous administration of oral spray at different concentrations (*v*/*v*). The severity of hepatocyte degeneration, necrosis/apoptosis, sinusoidal congestion, and inflammatory infiltration showed a dose-dependent increase. Scores were graded as “-” (no change), “+” (mild), “++” (moderate).

Histopathological Change	Control	1:1000 (*v*/*v*)	1:100 (*v*/*v*)	1:1 (*v*/*v*)	Neat (100%, *v*/*v*)
Hepatocyte degeneration	-	++	+	++	+
Necrosis/apoptosis	-	++	-	++	-
Interstitial edema	+	+	+	++	++
Inflammatory infiltration	-	-	-	-	++
Steatosis	-	-	-	-	-
Fibrosis	-	-	-	-	-

## Data Availability

The data presented in this study is available on request from the corresponding author.

## Abbreviations

The following abbreviations are used in this manuscript:

ATCC	American Type Culture Collection
CAM	Chorioallantoic Membrane
CFU	Colony Forming Unit
CMC	Carboxymethyl Cellulose
CO_2_	Carbon Dioxide
DAD	Diode Array Detector
EDD	Embryonic Development Day
EUCAST	European Committee on Antimicrobial Susceptibility Testing
H&E	Hematoxylin and Eosin
HPC	Hydroxypropyl Cellulose
HPLC	High Performance Liquid Chromatography
MC	Methyl Cellulose
MALDI-TOF MS	Matrix Assisted Laser Desorption Ionization Time of Flight Mass Spectrometry
MBC	Minimum Bactericidal Concentration
MIC	Minimum Inhibitory Concentration
NBF	Neutral Buffered Formalin
OD	Optical Density
PBS	Phosphate-Buffered Saline
RSD	Relative Standard Deviation
SD	Standard Deviation

## References

[1] Gao Z., Chen X., Wang C., Song J., Xu J., Liu X., Qian Y., Suo H. (2024). New strategies and mechanisms for targeting *Streptococcus mutans* biofilm formation to prevent dental caries: A review. Microbiol. Res..

[2] Korona-Glowniak I., Skawinska-Bednarczyk A., Wrobel R., Pietrak J., Tkacz-Ciebiera I., Maslanko-Switala M., Krawczyk D., Bakiera A., Borek A., Malm A. (2022). *Streptococcus sobrinus* as a predominant oral bacteria related to the occurrence of dental caries in polish children at 12 years old. Int. J. Environ. Res. Public Health.

[3] Beighton D. (2005). The complex oral microflora of high-risk individuals and groups and its role in the caries process. Community Dent. Oral Epidemiol..

[4] Valdez R.M.A., dos Santos V.R., Caiaffa K.S., Danelon M., Arthur R.A., Negrini T.d.C., Delbem A.C.B., Duque C. (2016). Comparative in vitro investigation of the cariogenic potential of bifidobacteria. Arch. Oral Biol..

[5] Poppolo Deus F., Ouanounou A. (2022). Chlorhexidine in Dentistry: Pharmacology, Uses, and Adverse Effects. Int. Dent. J..

[6] James P., Worthington H.V., Parnell C., Harding M., Lamont T., Cheung A., Whelton H., Riley P. (2017). Chlorhexidine mouthrinse as an adjunctive treatment for gingival health. Cochrane Database Syst. Rev..

[7] Eley B.M. (1999). Antibacterial agents in the control of supragingival plaque—A review. Br. Dent. J..

[8] Joosstens M., Valkenburg C., Van der Weijden F. (2025). Chemical agents to control biofilm formation in step 1 of care—Toothpastes and mouthwashes/concepts and challenges. Periodontol. 2000.

[9] Georgieva R., Yocheva L., Tserovska L., Zhelezova G., Stefanova N., Atanasova A., Danguleva A., Ivanova G., Karapetkov N., Rumyan N. (2015). Antimicrobial activity and antibiotic susceptibility of Lactobacillus and Bifidobacterium spp. intended for use as starter and probiotic cultures. Biotechnol. Biotechnol. Equip..

[10] Dumitrel S.-I., Matichescu A., Dinu S., Buzatu R., Popovici R., Dinu D.C., Bratu D.C. (2024). New Insights Regarding the Use of Relevant Synthetic Compounds in Dentistry. Molecules.

[11] Alparslan L., Giray B., Gulec M., Kaya N., Uvey D., Olgun A. (2023). Antibacterial and anthelmintic effect of the combination of pomegranate peel and olive leaf extracts. ACTA Pharm. Sci..

[12] de Oliveira N.M., Machado J., Chéu M.H., Lopes L., Criado M.B. (2024). Therapeutic Potential of Olive Leaf Extracts: A Comprehensive Review. Appl. Biosci..

[13] Bisignano G., Tomaino A., Lo Cascio R., Crisafi G., Uccella N., Saija A. (1999). On the in-vitro antimicrobial activity of oleuropein and hydroxytyrosol. J. Pharm. Pharmacol..

[14] Golestannejad Z., Khozeimeh F., Abtahi R., Zarei Z., Sadeghalbanaei L., Sadeghian R. (2020). Inhibitory effects of ethanolic, methanolic, and hydroalcoholic extracts of olive (*Olea europaea*) leaf on growth, acid production, and adhesion of *Streptococcus mutans*. Dent. Res. J..

[15] Nazzaro F., Fratianni F., Coppola R. (2013). Quorum Sensing and Phytochemicals. Int. J. Mol. Sci..

[16] Takó M., Kerekes E.B., Zambrano C., Kotogán A., Papp T., Krisch J., Vágvölgyi C. (2020). Plant Phenolics and Phenolic-Enriched Extracts as Antimicrobial Agents against Food-Contaminating Microorganisms. Antioxidants.

[17] Otero D., Oliveira F., Lorini A., Antunes B., Oliveira R., Zambiazi R. (2020). Oleuropein: Methods for extraction, purifying and applying. Rev. Ceres.

[18] Chastel T., Filiberti S., Mitola S., Ronca R., Turtoi A., Corsini M. (2025). Protocol for performing angiogenic and tumorigenic assays using the in ovo chick embryo chorioallantoic membrane model. STAR Protoc..

[19] Ribeiro L.N.M., Schlemper A.E., da Silva M.V., Fonseca B.B. (2022). Chicken embryo: A useful animal model for drug testing?. Eur. Rev. Med. Pharmacol. Sci..

[20] Alparslan L., Torkay G., Bal-Öztürk A., Köksal Karayıldırım Ç., Özdemir S. (2025). Smart Thermoresponsive Sol–Gel Formulation of Polyhexanide for Rapid and Painless Burn and Wound Management. Polymers.

[21] Bal-Öztürk A., Torkay G., İdil N., Akar R.O., Özbaş Z., Özkahraman B. (2024). Propolis-loaded photocurable methacrylated pullulan films: Evaluation of mechanical, antibacterial, biocompatibility, wound healing and pro-angiogenic abilities. Int. J. Biol. Macromol..

[22] Nejati O., Torkay G., Girgin A., Zaman B.T., Akar R.O., Giray B., Ulukaya E., Bakırdere S., Bal-Öztürk A. (2025). Biocompatible silver nanoparticles from apricot kernel skin: A green synthesis approach to antibacterial and antiangiogenic therapies. Chem. Pap..

[23] Bürgermeister J., Paper D.H., Vogl H., Linhardt R.J., Franz G. (2002). LaPSvS1, a (1→3)-beta-galactan sulfate and its effect on angiogenesis in vivo and in vitro. Carbohydr. Res..

[24] Bancroft J.D., Gamble M. (2008). Theory and Practice of Histological Techniques.

[25] Cho W.-Y., Kim D.-H., Lee H.-J., Yeon S.-J., Lee C.-H. (2020). Journal of Food Quality Evaluation of Effect of Extraction Solvent on Selected Properties of Olive Leaf Extract. J. Food Qual..

[26] Ramírez E.M., Brenes M., Romero C., Medina E. (2023). Olive Leaf Processing for Infusion Purposes. Foods.

[27] Majrashi T.A., El Hassab M.A., Mahmoud S.H., Mostafa A., Wahsh E.A., Elkaeed E.B., Hassan F.E., Eldehna W.M., Abdelgawad S.M. (2024). In vitro biological evaluation and in silico insights into the antiviral activity of standardized olive leaves extract against SARS-CoV-2. PLoS ONE.

[28] Nediani C., Ruzzolini J., Romani A., Calorini L. (2019). Oleuropein, a Bioactive Compound from *Olea europaea* L., as a Potential Preventive and Therapeutic Agent in Non-Communicable Diseases. Antioxidants.

[29] Pereira A.P., Ferreira I.C., Marcelino F., Valentão P., Andrade P.B., Seabra R., Estevinho L., Bento A., Pereira J.A. (2007). Phenolic Compounds and Antimicrobial Activity of Olive (*Olea europaea* L. *Cv*. Cobrançosa) Leaves. Molecules.

[30] Romero-Márquez J.M., Navarro-Hortal M.D., Forbes-Hernández T.Y., Varela-López A., Puentes J.G., Pino-García R.D., Sánchez-González C., Elio I., Battino M., García R. (2023). Exploring the Antioxidant, Neuroprotective, and Anti-Inflammatory Potential of Olive Leaf Extracts from Spain, Portugal, Greece, and Italy. Antioxidants.

[31] De Rossi L., Rocchetti G., Lucini L., Rebecchi A. (2025). Antimicrobial Potential of Polyphenols: Mechanisms of Action and Microbial Responses—A Narrative Review. Antioxidants.

[32] Guo W., Xu Y., Yang Y., Xiang J., Chen J., Luo D., Xie Q. (2023). Antibiofilm Effects of Oleuropein against Staphylococcus aureus: An In Vitro Study. Foods.

[33] Brookes Z.L.S., Bescos R., Belfield L.A., Ali K., Roberts A. (2020). Current uses of chlorhexidine for management of oral disease: A narrative review. J. Dent..

[34] Benavente-García O., Castillo J., Lorente J., Ortuño A., Del Rio J.A. (2000). Antioxidant activity of phenolics extracted from *Olea europaea* L. leaves. Food Chem..

[35] Esfandiary M.A., Khosravi A.R., Asadi S., Nikaein D., Hassan J., Sharifzadeh A. (2024). Antimicrobial and anti-biofilm properties of oleuropein against Escherichia coli and fluconazole-resistant isolates of Candida albicans and Candida glabrata. BMC Microbiol..

[36] Chen L., Wang S., Feng Y., Zhang J., Du Y., Zhang J., Ongeval C.V., Ni Y., Li Y. (2021). Utilisation of Chick Embryo Chorioallantoic Membrane as a Model Platform for Imaging-Navigated Biomedical Research. Cells.

[37] Durmus E., Inan O., Celik I., Sur E., Ozkan Y., Acar A., Aydin M.F. (2005). Use of the fertilized hen’s egg in the evaluation of embryotoxicity of dental alloys. J. Biomed. Mater. Res. Part B Appl. Biomater..

[38] Khosravi A., Sharifi I., Tavakkoli H., Derakhshanfar A., Keyhani A.R., Salari Z., Mosallanejad S.S., Bamorovat M. (2018). Embryonic toxico-pathological effects of meglumine antimoniate using a chick embryo model. PLoS ONE.

[39] Karygianni L., Cecere M., Argyropoulou A., Hellwig E., Skaltsounis A.L., Wittmer A., Tchorz J.P., Al-Ahmad A. (2019). Compounds from *Olea europaea* and *Pistacia lentiscus* inhibit oral microbial growth. BMC Complement. Altern. Med..

[40] Taskan M.M., Balci Yuce H., Karatas O., Gevrek F., Toker H. (2019). Evaluation of the effect of oleuropein on alveolar bone loss, inflammation, and apoptosis in experimental periodontitis. J. Periodontal Res..

